# Effects of Sodium-Glucose Cotransporter-2 Inhibitors and Thiazolidinedione on New-Onset Atrial Fibrillation Risk to Patients with Type 2 Diabetes

**DOI:** 10.31083/j.rcm2309303

**Published:** 2022-09-09

**Authors:** Haegeun Song, Yoo Ri Kim, Seung Eun Lee, Hyewon Nam, Hoseob Kim, Dae-Sung Kyoung, Kyoung-Ah Kim

**Affiliations:** ^1^Division of Cardiology, Department of Internal Medicine, Chung-Ang University, Gwang-Myong Hospital, 14353 Seoul, Republic of Korea; ^2^Division of Cardiology, Department of Internal Medicine, Chonnam National University, 61469 Gwangju, Republic of Korea; ^3^Division of Endocrinology, Department of Internal Medicine, Dongguk University Ilsan Hospital, 10326 Goyang, Republic of Korea; ^4^Data Science Team, Hanmi Pharm. Co. Ltd, 05545 Seoul, Republic of Korea

**Keywords:** sodium-glucose cotransporter-2 inhibitors, atrial fibrillation, thiazolidinediones, diabetes mellitus type 2

## Abstract

**Background and Objectives:**

Type 2 diabetes (T2D) is an independent risk factor for the development of atrial fibrillation (AF). Sodium-glucose cotransporter-2 inhibitors (SGLT-2i) have recently been shown to decrease the incidence of AF through several mechanisms, including the reduction of atrial dilatation via diuresis and the lowering of body weight. In observational studies of diabetic patients, the use of thiazolidinedione (TZD) was found to have a protective effect on new-onset AF. In this study, we aimed to compare the effect of SGLT-2i and TZD on the risk of AF in patients with T2D.

**Methods:**

We enrolled 69,122 patients newly prescribed SGLT-2i and 94,262 patients prescribed TZD from January 2014 to December 2018, using the Korean National Health Insurance Service database. We compared new-onset AF events (hospitalizations and outpatient events) in SGLT-2i and TZD groups after having taken medication for greater than 90 days.

**Results:**

During a mean follow-up of 1.8 years, 397 (0.72%) new-onset AF events occurred in the SGLT-2i group and 432 (0.79%) events in the TZD group following propensity score matching (each group n = 54,993). The hazard ratio (HR) of AF was 0.918 (95% confidence interval: 0.783–1.076, *p* = 0.29) in SGLT-2i-treated patients compared with TZD-treated patients.

**Conclusions:**

In this study, the risk of new-onset AF is comparable in patients treated with SGLT-2i and TZD in T2D. Either SGLT-2i or TZD would be a reasonable choice for T2D patients who are at risk for AF.

## 1. Introduction

Atrial fibrillation (AF) is the most common arrhythmia in clinical practice and is considered an independent risk factor for death, with a twofold increased risk of all-cause mortality in women and a 1.5-fold increase in men, with an overall 3.5-fold mortality risk increase due to heart failure (HF), malignancy, infection and embolic cerebrovascular disease [1, 2, 3].

Type 2 diabetes is generally known to be an independent risk factor for AF. A meta-analysis of several cohort and case-control studies reported that patients with type 2 diabetes had a 34% greater risk of developing AF than the general population [4]. The pathophysiology of diabetes-related AF is not fully understood, but it is associated with structural and electrical remodelling and autonomic dysfunction in the atria as a result of glycemic fluctuation, oxidative stress and inflammation [5]. It is known that glucose-lowering drugs have differing effects on the incidents of AF. In general, glucose-lowering drugs that cause hypoglycemia are known to increase the risk of AF [5].

In observational studies, thiazolidinedione (TZD), an insulin sensitizer, has shown a protective effect against the development of new-onset AF. This may be because of its pleiotropic effects, such as anti-inflammatory and antioxidant properties [6].

A new class of oral hypoglycemic agents, sodium-glucose cotransporter-2 inhibitors (SGLT-2i), has been shown to potentially reduce the risk of cardiovascular outcomes in terms of HF and all-cause mortality [7, 8]. SGLT-2i has also shown benefit in reducing the relative risk of AF in patients with type 2 diabetes [9, 10]. The mechanism of this favorable outcome is incompletely understood, but it has been suggested that increased renal glucose excretion induces additional osmotic diuresis, leading to decreased arterial blood pressure, improved myocardial efficiency, delayed myocardial structural remodeling, and decreased atrial dilation [11].

Therefore, we designed this study to compare the effects that SGLT-2i and TZD has on the risk of AF in patients with type 2 diabetes.

## 2. Methods

This study used the National Health Insurance Service (NHIS) database, established by the Korean NHIS. In order to access the NHIS database, an application form for a research proposal must be submitted to the NHIS Korean Committee of Research Support. After a review and approval, data are made available in a deidentified format. This study was also approved by the Institutional Review Board of Dongguk University Ilsan Hospital (No.: 2019-02-002-002). Written informed consent was waived by the Institutional Review Board. All methods in this study were performed in accordance with the Declaration of Helsinki.

The NHIS in Korea is a single-payer healthcare system, mandatory for all residents of Korea. The NHIS established a national health information database which includes patient demographic information, medical claims, medications, health checkups and death information. Because the NHIS provides regular cost-free health checkups, which include a physical examination, blood tests, and urine tests to all applicable examinees, these results can be integrated with other medical information so that comprehensive and detailed analyses are possible.

### 2.1 Study Population

Because SGLT-2i has been available since 2014 in Korea, we included patients from January 2014 to December 2018 who were new users of either SGLT-2i or TZD. We defined type 2 diabetes as the presence of ICD-10 code (E11-14). We defined a new user as any patient who had received any SGLT-2i (dapagliflozin, empagliflozin, ipragliflozin, or ertugliflozin) or TZD (pioglitazone or lobeglitazone) between January 2014 and December 2018, with a one-year washout period. Among the new users, we only included those who had been prescribed study drugs for 90 or more days. Diagnostic codes of patients from January 2008 to December 2013 were collected and combined to determine the baseline characteristics of enrolled patients.

**Fig. 1.**
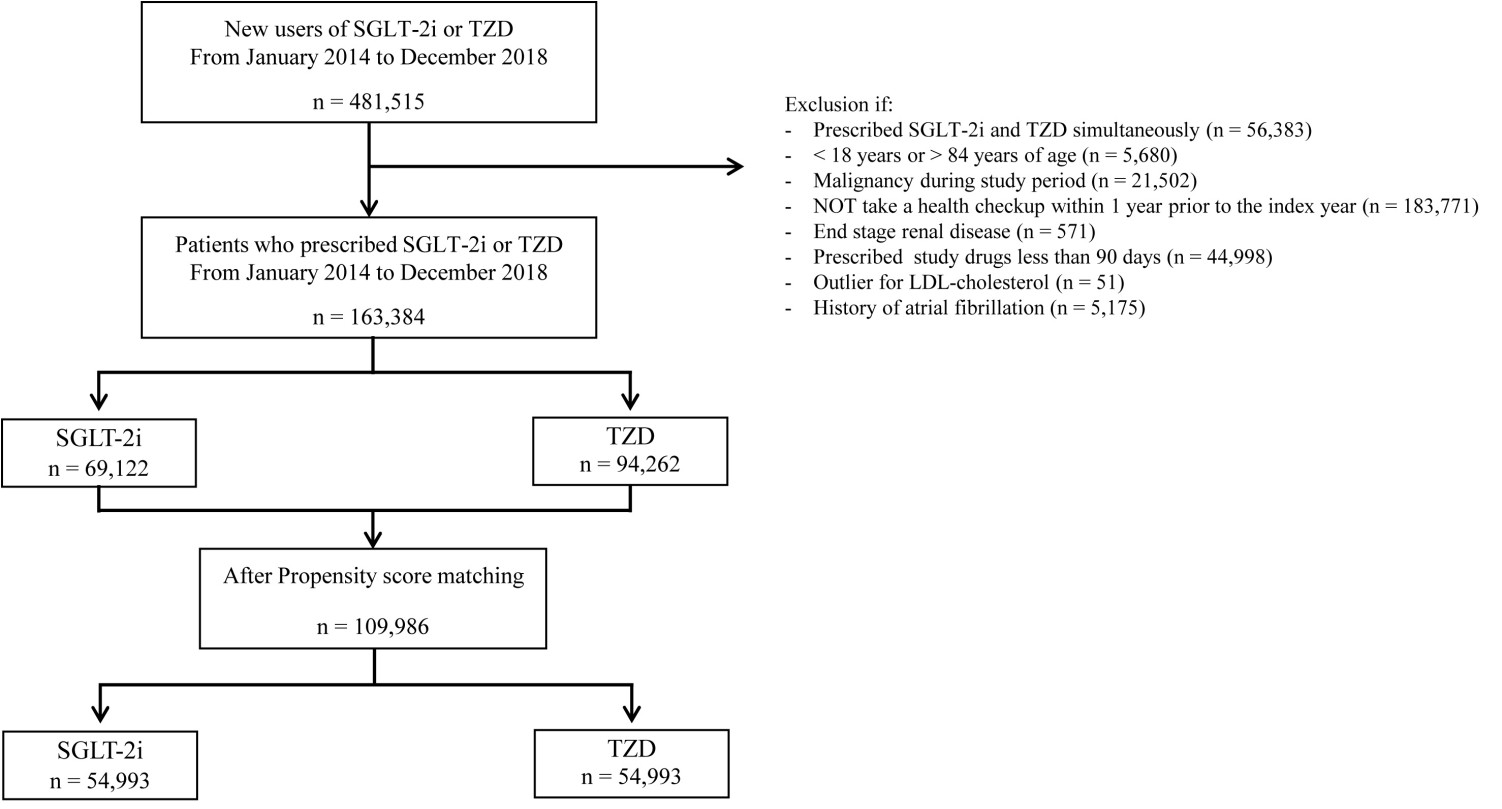
**Flow chart of study population**. LDL, low-density lipoprotein; SGLT-2i, sodium-glucose cotransporter-2 inhibitor; TZD, thiazolidinedione.

We identified a total of 481,515 new users of SGLT-2i or TZD. Index year was the year the drug of interest started. We excluded patients who has a history of AF or had been prescribed study drugs for fewer than 90 days or patients who had been prescribed SGLT-2i and TZD simultaneously. We also excluded patients younger than 18 or older than 84 years, patients who had malignancy, end-stage renal disease, or patients who had not had a health checkup within one year prior to the index year. Fifty-one individuals with extremely high low-density lipoprotein (LDL)-cholesterol levels (≥300 mg/dL) were excluded due to possible familial hypercholesterolemia. We identified a total of 163,384 patients, of which 69,122 used SGLT-2i and 94,262 used TZD. After propensity matching, a total of 109,986 patients were identified, 54,993 in each group (Fig. 1). Participants were followed until the outcome event, death, or 31 December 2018 whichever came first.

### 2.2 Demographic Factors at Baseline

During regular health checks, all participants were asked to fill out questionnaires including questions about smoking status, alcohol consumption, and physical activity. If the patients smoked 100 cigarettes or more during their lifetime and continued smoking, we defined them as current smokers. We defined heavy drinkers as those who drank five or more days per week. We defined patients who exercise vigorously three or more days a week or moderately five or more days a week as being physically active.

Body mass index (BMI) was calculated as weight divided by height squared (${\mathrm{kg/m}}^{2}$). Venous sampling was done at least eight hours after fasting to examine fasting blood sugar (FBS), total cholesterol, triglycerides, high-density lipoprotein (HDL) cholesterol, LDL cholesterol, aspartate aminotransferase, alanine aminotransferase, gamma-glutamyl transferase (GGT), and creatinine levels. Estimated glomerular filtration rate (eGFR) was calculated using the Chronic Kidney Disease Epidemiology Collaboration equation [12]. Proteinuria was defined as being 1+ or greater by urine protein dipstick test.

### 2.3 Baseline Comorbidities

We defined hypertension using the ICD-10 code for hypertension (I10–13, I15) together with antihypertensive medications. Those individuals who had a systolic blood pressure of 140 mmHg or greater and/or a diastolic blood pressure of 90 mmHg or greater on health checkup were also defined as having hypertension. The ICD-10 code of dyslipidemia (E78) with the administration of lipid-lowering agents or a total cholesterol level of 240 mg/dL or greater was used for the diagnosis of dyslipidemia.

Subjects with diagnostic codes for stroke (I60–I64), myocardial infarction (MI) (I21–I23), or unstable angina (I20) at least once were defined as having each disease respectively. Peripheral artery disease was diagnosed when patients had two or more outpatient or one or more inpatient diagnoses of ICD-10 codes I70 and I73. If patients had any of these four diseases, we defined them as having had prior cardiovascular disease (CVD).

HF was diagnosed if patients had had two or more outpatient diagnoses or one or more inpatient diagnoses of ICD-10 codes for HF (I11.0, I13.0, I13.2, I50), together with relevant medications including spironolactone, loop diuretics (furosemide, torsemide), beta blockers (carvedilol, bisoprolol, nebivolol, metoprolol) or sacubitril/valsartan.

### 2.4 Outcome

The primary outcome of this study is the diagnosis of new-onset AF following the prescription of study drugs at least 90 days. AF patients were defined as those with one or more diagnoses at the time of hospital discharge or at the outpatient clinic (ICD-10 code I48). In addition, we examined new-onset AF having a concurrent diagnosis of hospitalization for HF (hHF) or AF with concurrent hospitalization for stroke. AF with concurrent hHF was defined if patients had AF and a main diagnosis of hospitalization for HF (I11.0, I13.0, I13.2, I50) regardless of temporal association. AF concurrent with hospitalization for stroke was defined if patients had AF and were hospitalized with a main diagnosis of stroke (I60–I64).

### 2.5 Statistical Analyses

We conducted a propensity score matched analysis to minimize differences between the two treatment groups. Variables used to match the study population include age, sex, stroke, MI, peripheral artery disease, unstable angina, hypertension, dyslipidemia, heart failure, angiotensin converting enzyme inhibitor, angiotensin receptor blocker, beta blocker, statin, antiplatelet, anticoagulant, smoking, drinking, proteinuria, weight, height, waist circumference, systolic blood pressure, diastolic blood pressure, hemoglobin, FBS, HDL-cholesterol, LDL-cholesterol, triglyceride, aspartate aminotransferase, alanine aminotransferase (ALT), GGT, eGFR, and index year. Allocation with 1:1 ratio was performed using a greedy method. After then, we evaluated matching quality using absolute standardized difference (ASD) in mean between the two groups [13]. An ASD of less than 0.1 was considered to be negligible for each covariate.

After propensity score matching, Kaplan-Meier curves and log-rank tests were used to compare the cumulative incidence of outcomes according to treatment group. If there were more than two incidents in any patient, only the outcome of the first incident was included. The incidence rate of outcomes was expressed as the number of events per 1000 person-years. The risk of AF was obtained using Cox proportional hazards regression analyses. And we performed subgroup analysis to define the effect of sex, age (<65, 65–74, ≥75 yrs), prior CVD, prior HF, renal function (eGFR <60, ≥60 mL/min/1.73 $m^{2}$), and fasting glucose (<130, 130–159, ≥160 mg/dL) for the incidence of new-onset AF.

If drug crossover had occurred (e.g., SGLT-2i → TZD or TZD → SGLT-2i), as-treated analysis was applied. Patients were followed until either an outcome event, death, or 31 December 2018, whichever occurred first.

After analyses, sample size calculation was performed to prove the relevance of study results. Sample size and power calculation for survival non-inferiority were performed using nQuery Advisor 7.0 (Statistical Solutions Ltd., Cork, Ireland). Given the type I error α = 0.025 and power of 1-β = 80%, we assumed the hazard ratio between SGLT-2i and TZD defining non-inferiority was set to 1.2. The total sample size of 80,684 participants was required to test for survival non-inferiority. All statistical analyses were performed using SAS version 9.4 (SAS Institute, Cary, NC, USA). *p* values of <0.05 were considered statistically significant.

## 3. Results

### 3.1 Baseline Characteristics

A total of 69,122 patients treated with SGLT-2i and 94,262 patients with TZD were identified. Before propensity matching, SGLT-2i users were younger and more obese than those who used TZD (**Supplementary Table 1**). They had higher prevalence of dyslipidemia and the mean level of ALT was also higher in those treated with SGLT-2i. SGLT-2i users were more likely to have eGFR ≥60 mL/min/1.73 $m^{2}$. After propensity matching, each group included 54,993 patients, and their mean age was 57.0 years, with men being 58% and prior CVD being 18.4%. The mean BMI of matched cohorts was 26.3 ${\mathrm{kg/m}}^{2}$ and mean FBS was 151.6 mg/dL. Ninety-two percent of patients had an eGFR equal to or greater than 60 mL/min/1.73 $m^{2}$, and proteinuria was 12.1%. Overall, baseline characteristics of matched cohorts were well-balanced with ASD less than 0.1 for all variables (Table 1).

**Table 1.** **Baseline characteristics of study population after propensity matching**.

		SGLT-2i	TZD	ASD
		(n = 54,993)	(n = 54,993)	
Men		31,530 (57.3)	31,954 (58.1)	0.0157
Age, yrs		56.5 (10.5)	57.6 (10.5)	0.0295
	≥65	12,682 (23.1)	13,388 (24.3)	
	<65	42,311 (76.9)	41,605 (75.7)	
Prior CVD		10,147 (18.5)	10,064 (18.3)	0.0039
	Stroke	5024 (9.1)	5154 (9.4)	0.0080
	MI	1993 (3.6)	1905 (3.5)	0.0087
	PAD	1255 (2.3)	1277 (2.3)	0.0027
	Unstable angina	4054 (7.4)	3915 (7.1)	0.0099
Comorbidities				
	Hypertension	37,976 (69.1)	37,791 (68.7)	0.0073
	Dyslipidemia	35,974 (65.4)	35,738 (65.0)	0.0090
	HF	2975 (5.4)	2861 (5.2)	0.0092
Medication use				
	ARB	32,222 (58.6)	31,958 (58.1)	0.0098
	ACEi	6224 (11.3)	6258 (11.4)	0.0019
	BB	18,073 (32.9)	17,802 (32.4)	0.0105
	Statin	34,773 (63.2)	34,606 (62.9)	0.0063
	Anti-platelet	27,410 (49.8)	27,653 (50.3)	0.0088
	Anti-coagulant	582 (1.1)	592 (1.1)	0.0018
Current smoker		12,764 (23.2)	12,854 (23.4)	0.0039
Heavy drinker		2363 (4.3)	2440 (4.4)	0.0068
Physically active		12,065 (21.9)	12,019 (21.9)	0.002
Height, cm		163.6 (9.1)	163.5 (9.4)	0.0075
Weight, kg		70.9 (13.1)	70.4 (13.5)	0.0419
BMI, ${\mathrm{kg/m}}^{2}$		26.4 (3.8)	26.2 (3.8)	0.0649
	≥25	34,697 (63.1)	32,966 (59.9)	
	<25	20,296 (36.9)	22,027 (40.1)	
WC, cm		88.1 (9.4)	88 (10.3)	0.0280
	men, ≥90; women, ≥80	32,778 (59.6)	32,023 (58.2)	
	men, <90; women, <80	22,215 (40.4)	22,970 (41.8)	
SBP, mmHg		127 (14.6)	126.9 (14.3)	0.0027
DBP, mmHg		77.7 (9.7)	77.6 (9.5)	0.0101
FBS, mg/dL		151.4 (52.2)	151.9 (50.2)	0.0100
Triglycerides*		156.9 (96.5)	155.4 (94.3)	0.0154
HDL-cholesterol*		50.4 (12.8)	50.5 (14.4)	0.0091
LDL-cholesterol*		93.5 (40.8)	92.7 (41.1)	0.0191
AST*		30.6 (22.4)	30.3 (20.9)	0.0136
ALT*		34.2 (27.4)	33.7 (26.5)	0.0205
GGT*		48.9 (59.4)	49.4 (61.1)	0.0070
Hemoglobin, g/dL		14.5 (1.6)	14.4 (1.6)	0.0376
eGFR, mL/min/1.73 $m^{2}$		89.9 (24.8)	89.4 (29.1)	0.0154
	≥60	51,316 (93.3)	50,646 (92.1)	
	<60	3677 (6.7)	4347 (7.9)	
Proteinuria		6695 (12.2)	6679 (12.1)	0.0009

Results are expressed as means (SDs) for continuous variables and frequencies and percentage relative frequencies for categorical variables. *The log-transformation was used to compare the means of SGLT-2i users and TZD-users. ACEi, angiotensin-converting enzyme inhibitor; ALT, alanine aminotransferase; ARB, angiotensin II receptor blocker; ASD, absolute standardized difference; AST, aspartate aminotransferase; BB, beta blocker; BMI, body mass index; CVD, cardiovascular disease; DBP, diastolic blood pressure; eGFR, estimated glomerular filtration rate; FBS, fasting blood sugar; GGT, gamma glutamyl transferase; HDL, high-density lipoprotein; HF, heart failure; LDL, low-density lipoprotein; MI, myocardial infarction; PAD, peripheral artery disease; SBP, systolic blood pressure; SGLT-2i, sodium-glucose co-transporter-2 inhibitor; TZD, thiazolidinedione; WC, waist circumference.

### 3.2 Risks of New-Onset of AF with SGLT-2i versus TZD 

**Fig. 2.**
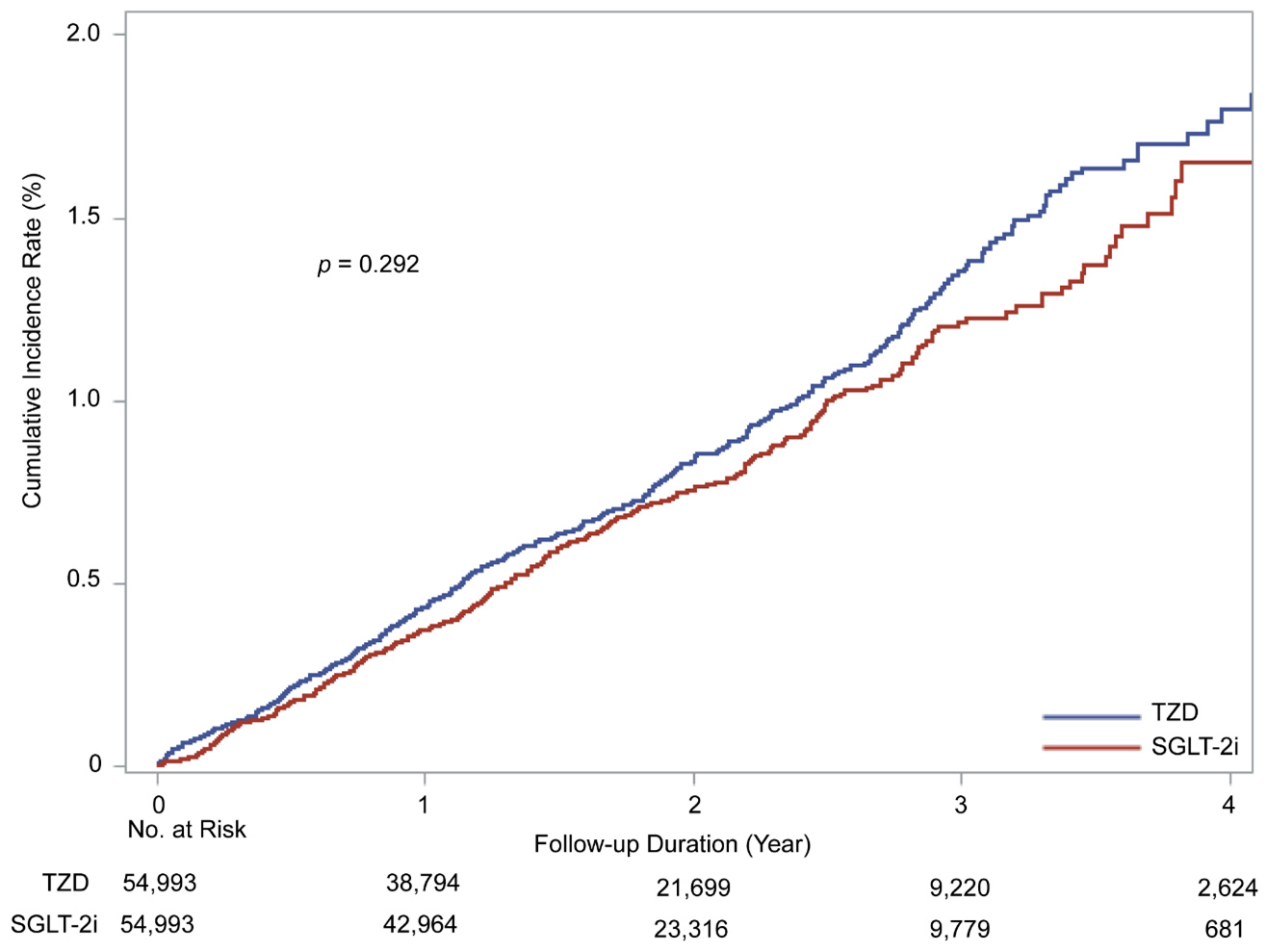
**Cumulative incidence of AF in the SGLT-2i and TZD groups**. SGLT-2i, Sodium-glucose cotransporter-2 inhibitors; TZD, Thiazolidinedione.

**Fig. 3.**
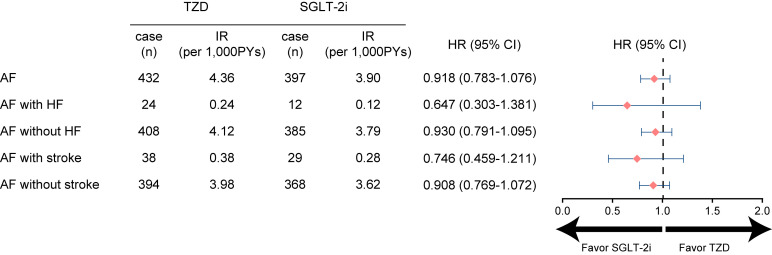
**Risk of AF in SGLT-2i and TZD groups**. AF was classified into AF with or without HF and stroke to investigate the effect of study drugs on HF and hard outcome, respectively. AF, atrial fibrillation; CI, confidence interval; HF, heart failure; IR, incidence rate; HR, hazard ratio; SGLT-2i, sodium-glucose cotransporter-2 inhibitors; TZD, thiazolidinedione.

The mean follow-up duration time was 657.5 days for the TZD group and 675.9 days for the SGLT-2i group. During the follow-up, 829 patients were diagnosed with newly developed AF (432 in the TZD group, 397 in the SGLT-2i group). The incidence rate of new-onset AF was 4.36 and 3.90 per 1000 person-years for the TZD and SGLT-2i groups respectively. Cumulative incidence of new-onset AF did not differ significantly between groups (log-rank test, *p* = 0.292) (Fig. 2). In the SGLT-2i group, HR from new-onset AF was 0.918 (95% CI, 0.783–1.076) as compared to the TZD group (Fig. 3).

### 3.3 The Risk of New-Onset AF Concurrent with hHF or Hospitalization for Stroke 

We examined the risk of AF coexisting with HF since they frequently occur together [14] and SGLT-2i is known to reduce HF risk. Among 829 individuals with new-onset AF, 4.3% had incidentally concurrent HF regardless of temporal association. The incidence rate of new-onset AF concurrent with hHF was 0.24 and 0.12 per 1000 person-years for the TZD and SGLT-2i groups respectively (Fig. 3). The HR of new-onset AF concomitant with hHF was 0.647 (95% CI, 0.303–1.381) in the SGLT-2i group compared to the TZD group. To evaluate whether AF-related hard outcomes differ between groups, we compared the risk of AF concurrent with hospitalization for stroke. Among 829 individuals with new-onset AF, 8.1% had a concurrent stroke regardless of temporal association. The incidence rate of concurrent new-onset AF with hospitalization for stroke was 0.38 and 0.28 per 1000 person-years for the TZD and SGLT-2i groups respectively (Fig. 3). The HR of new-onset AF concurrent with hospitalization for stroke was 0.746 (95% CI, 0.459–1.211) in the SGLT-2i group as compared to the TZD group.

### 3.4 Subgroup Analyses

**Fig. 4.**
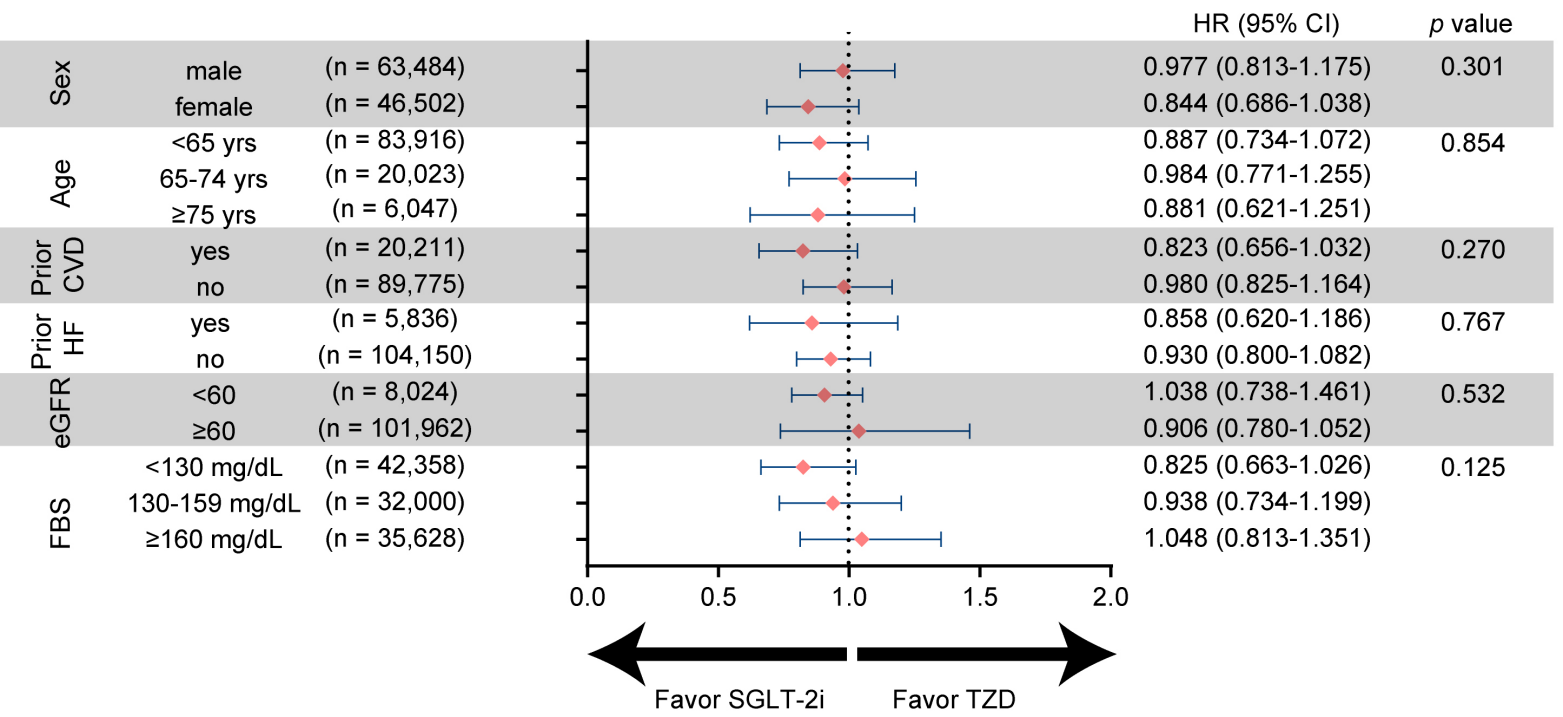
**Risk of AF according to subgroups in SGLT-2i and TZD**. Subgroup analyses to investigate whether effects of drugs differ between subgroups of study population. Event rates were calculated as the number of events divided by the total number of populations in the group. AF, atrial fibrillation; CI, confidence interval; CVD, cardiovascular disease; HF, heart failure; HR, hazard ratio; eGFR, estimated glomerular filtration rate; FBS, fasting blood sugar; SGLT-2i, sodium-glucose cotransporter-2 inhibitors; TZD, thiazolidinedione.

We performed stratified analyses according to subgroups of age, sex, prior CVD, prior HF, eGFR level, and FBS level; Variables showed no difference in treatment effects between the groups receiving the two drugs. However, among patients with lower fasting blood glucose levels, those treated with SGLT-2i tended to have a lesser risk of AF, although statistical significance was not achieved (*p* interaction = 0.125; Fig. 4).

## 4. Discussion

In this retrospective cohort study using the NHIS database of Korea, we found no differences in the incidence of new-onset AF between type 2 diabetes patients treated with SGLT-2i and TZD. The SGLT-2i-treated patients tend to be associated with lower AF risk concurrent with hHF, although this did not reach statistical significance.

Recently published meta-analysis with 16 randomized control trials demonstrated that SGLT-2i (empagliflozin 4 studies, canagliflozin 6 studies, dapagliflozin 6 studies) had great benefits in reducing the risk of AF/AFL (atrial flutter) in type 2 diabetes populations [9]. These benefits were not affected by age, body weight, glycosylated haemoglobin A1c (HbA1c), or systolic blood pressure. In the study, dapagliflozin was associated with a significant reduction of AF/AFL, whereas canagliflozin and empagliflozin had no effect on reducing AF/AFL events. A subgroup analysis of the DECLARE-TIMI 58 trial of patients with type 2 diabetes also found that dapagliflozin reduced the risk of AF/AFL events during follow-up by 19%, as well as the number of AF/AFL events overall. These reductions were consistent across major subgroups, including sex, presence of atherosclerotic cardiovascular disease, history of AF/AFL, history of HF, history of ischemic stroke, HbA1c, body mass index, blood pressure, or eGFR, which are well-known to be associated with the risk of AF/AFL [10]. Although many studies have been conducted, it is still unclear how SGLT-2i affects AF. Considering that there is close relationship between cardiovascular impairment and renal dysfunction which worsen each other [15], the mechanism of SGLT-2i on AF may be that it mediates natriuresis and glucosuria, thus lowering cardiac preload and reducing pulmonary congestion and systemic edema, allowing for proportionally greater reductions of interstitial volume, as compared to intravascular volume [16]. This reduces sympathetic nervous system overdrive [17], oxidative stress, inflammation [18] and vascular stiffness [19]. The cardio-renal protective effect of SGLT-2i was confirmed in many recent clinical trials. Cardiovascular outcome trials with SGLT-2i in type 2 diabetes showed unprecedented outcomes in the prevention of worsening heart failure, renal disease progression and mortality, further proved by randomized controlled trials in patients with heart failure and chronic kidney disease, with or without diabetes [20].

TZDs, the insulin-sensitizing agents, are known to have a strong protective effect on atherosclerosis-driven events such as cardiac or cerebrovascular disease [21]. Zhang *et al*. [6] conducted a meta-analysis of 130,854 diabetic patients in seven studies to evaluate TZD’s effect on AF development. This elucidated pioglitazone’s beneficial effect on reducing the risk of new-onset or recurrent AF by 30% [6]. Although pioglitazone might worsen HF and peripheral edema by inducing sodium-water retention, it positively modulates numerous cardiovascular functions (reduced inflammation of plaque, improved left ventricular systolic-diastolic function, improved arterial stiffness) and risk factors (blood pressure, blood lipid, adipose tissue physiology), and it shows meaningful reduction of major adverse cardiovascular events [22] and AF incidences [6].

In the present study, we compared the effect of SGLT-2i and TZD on new-onset AF, and there was no difference between the two groups, possibly because of the beneficial effect of both drugs. These results are consistent in patients, regardless of the presence or absence of HF at baseline. Of note, SGLT-2i-treated patients tend to have a lower risk of new-onset AF concurrent with hHF, although this does not reach statistical significance (HR 0.647, 95% CI, 0.303–1.381). The presence of either AF or HF increases the risk of developing the other [14]. AF results in atrial dilatation and left atrial volume overload that can promote HF, and HF can facilitate atrial remodeling, which makes for the development of AF [14]. Considering that SGLT-2i reduces the risk of hHF, SGLT-2i may help in reducing the incidence of AF and HF in those with severe HF that requires hospitalization. We saw no statistically significant difference in this study, possibly because the incidence of hHF was too small. Further studies with longer-term follow-ups are needed to verify this observation.

The current study found that SGLT-2i-treated group tended to have a lesser risk of AF in those with lower fasting glucose level, although statistical significance was not achieved. These findings may be explained by previous studies suggesting that glucose-lowering drugs may increase the risk of AF at FBS less than 130 mg/dL [23]. Previously, Lee *et al*. [24], reported that patients taking TZD have a higher risk of hypoglycemia than those without, and the patients taking TZD were more likely to have been prescribed three or more classes of antidiabetic drugs simultaneously in Korea. Thus, it is possible that glucose-lowering drugs that induce hypoglycemia were concomitantly used in the TZD group.

There are several limitations that should be considered in this study. First, this retrospective observational study using the claims databases has limitations. There were unmeasured confounding factors, such as HbA1c levels or duration of diabetes. Considering a recent meta-analysis that showed a 10% increased risk of AF per 20 mg/dL increase in blood glucose [25], potential effect of differential hyperglycemia between groups could not be excluded. Additionally, we could not evaluate specific clinical information such as HFpEF (heart failure with preserved ejection fraction) or HFrEF (heart failure with reduced ejection fraction), which are not included in the NHIS database. Second, discontinuation of study drugs or switching to other classes of drug could not be considered due to lack of data, when performing as-treated analysis. This might dilute the benefit of study drugs. Third, SGLT-2i was introduced in Korea in 2014, and its prescription rate was low. Therefore, the NHIS database had included fewer at-risk patients at the end of the follow-up period. Therefore, this short follow-up period is also a limitation. Forth, in Korea, SGLT-2i has been mainly used for younger obese patients with normal renal function [26]. Because we used a propensity score matched analysis to balance baseline characteristics between groups, we were unable to thoroughly examine the effects of drugs in patients at high risk for AF with renal dysfunction. Also, unfortunately, we couldn’t include elderly patients over 85 years of age in the study since the Korean Ministry of Food and Drug Safety issued a label warning to limit the use of SGLT-2i in this age group due to insufficient data on the volume depletion of SGLT-2i when it was introduced in 2014. Fifth, detection rate of AF could be lower than real AF incidence. To overcome this fundamental issue of AF detection, we enrolled relatively large number of patients and evaluated in a country where standardized in treatment and evaluation guided by government insurance system. Lastly, we only compared two drugs, TZD and SGLT-2i. Notably, population-based studies showed protective effect of metformin [27] and dipeptidyl peptidase 4 inhibitors [28] and harmful effect of sulfonylurea [29] in terms of new-onset AF. Regarding the effect of metformin on incident AF, we could not analyze it since more than 80% of patients with diabetes were using it as the first-line treatment in Korea [30].

If we had performed an analysis including other antidiabetic drugs, the effects would have been more obvious. Future studies with prospective design, longer-term follow-ups, and multiple comparators are needed to confirm the results of this study. Despite these limitations, to our knowledge, this is the first study to directly compare the effect of SGLT-2i and TZD in terms of new-onset AF outcomes using a large nationwide database.

## 5. Conclusions

In this study, the risk of new-onset AF was comparable in patients with type 2 diabetes treated with SGLT-2i and TZD in the national cohort. SGLT-2i or TZD would be a reasonable choice for patients with type 2 diabetes at risk of developing AF compared to other glucose-lowering drugs with a risk of hypoglycemia.

## Supplementary Material

Supplementary material associated with this article can be found, in the online version, at https://doi.org/10.31083/j.rcm2309303.
