# Supplementary material for: Effects of Sodium-Glucose Cotransporter-2 Inhibitors and Thiazolidinedione on New-Onset Atrial Fibrillation Risk to Patients with Type 2 Diabetes
Source: Rev Cardiovasc Med. 2022 Sep 9;23(9):303. doi: 10.31083/j.rcm2309303 (PMC11262332; doi:10.31083/j.rcm2309303)
Supplement: Supplementary file 1 [file 2153-8174-23-9-303-s1.docx]

Supplementary Table 1. Baseline characteristics of study population before propensity matching.

|  | | SGLT-2i (n =69,122) | TZD (n =94,262) | ASD |
| --- | --- | --- | --- | --- |
| Men | | 38,774 (56.1) | 57,038 (60.5) | 0.0896 |
| Age, yrs | | 55.5 (10.5) | 59.3 (10.7) | 0.2868 |
|  | ≥65 | 13,762 (19.9) | 30,526 (32.4) |  |
|  | <65 | 55,360 (80.1) | 63,736 (67.6) |  |
| Prior CVD | | 12,761 (18.5) | 17,676 (18.8) | 0.0075 |
|  | Stroke | 5864 (8.5) | 10,259 (10.9) | 0.0812 |
|  | MI | 2803 (4.1) | 2808 (3.0) | 0.0584 |
|  | PAD | 1521 (2.2) | 2184 (2.3) | 0.0078 |
|  | Unstable angina | 5431 (7.9) | 5907 (6.3) | 0.0621 |
| Comorbidities | |  |  |  |
|  | Hypertension | 48,287 (69.9) | 64,918 (68.9) | 0.0214 |
|  | Dyslipidemia | 46,069 (66.6) | 57,730 (61.2) | 0.1127 |
|  | HF | 4139 (6.0) | 4447 (4.7) | 0.0565 |
| Medication use | |  |  |  |
|  | ARB | 41,148 (59.5) | 54,782 (58.1) | 0.0287 |
|  | ACEi | 7745 (11.2) | 11,589 (12.3) | 0.0338 |
|  | BB | 23,472 (34.0) | 29,539 (31.3) | 0.0559 |
|  | Statin | 44,500 (64.4) | 55,877 (59.3) | 0.1051 |
|  | Anti-platelet | 33,657 (48.7) | 50,168 (53.2) | 0.0907 |
|  | Anti-coagulant | 734 (1.1) | 1004 (1.1) | 0.0003 |
| Current smoker | | 15,952 (23.1) | 22,457 (23.8) | 0.0176 |
| Heavy drinker | | 2890 (4.2) | 4363 (4.6) | 0.0218 |
| Physically active | | 14,985 (21.7) | 20,838 (22.1) | 0.0103 |
| Height, cm | | 163.9 (9.2) | 163 (9.3) | 0.0907 |
| Weight, kg | | 72.6 (14.1) | 68 (12.8) | 0.3415 |
| BMI, kg/m^2^ | | 26.9 (4.0) | 25.5 (3.6) | 0.3105 |
|  | ≥25 | 46,346 (67.0) | 49,004 (52.0) |  |
|  | <25 | 22,776 (33.0) | 45,258 (48.0) |  |
| WC, cm | | 89.1 (9.9) | 86.7 (9.7) | 0.2278 |
|  | men, ≥90; women, ≥80 | 43,765 (63.3) | 49,143 (52.1) |  |
|  | men, <90; women, <80 | 25,357 (36.7) | 45,119 (47.9) |  |
| SBP, mmHg | | 126.9 (14.5) | 127.2 (14.7) | 0.0164 |
| DBP, mmHg | | 78 (9.7) | 77.2 (9.6) | 0.0871 |
| FBS, mg/dL | | 150 (50.9) | 154.1 (54.8) | 0.0772 |
| Triglycerides^*^ | | 159.7 (95.9) | 150.3 (92.3) | 0.0994 |
| HDL-cholesterol^*^ | | 50.3 (12.7) | 50.6 (14.3) | 0.0261 |
| LDL-cholesterol^*^ | | 93.1 (42.0) | 93.8 (40.0) | 0.0167 |
| AST^*^ | | 31.1(22.7) | 29.2 (23.5) | 0.0823 |
| ALT^*^ | | 35.6 (28.9) | 31.2 (25.7) | 0.1620 |
| GGT^*^ | | 50.2 (59.2) | 46.7 (63.0) | 0.0582 |
| Hemoglobin, g/dL | | 14.6 (1.6) | 14.0 (1.7) | 0.3614 |
| eGFR, mL/min/1.73 m^2^ | | 91.1 (27.1) | 86.2 (27.6) | 0.1802 |
|  | ≥60 | 64,974 (94.0) | 83,544 (88.6) |  |
|  | <60 | 4148 (6.0) | 10,718 (11.4) |  |
| Proteinuria | | 8343 (12.1) | 12,272 (13.0) | 0.0287 |

Results are expressed as means (SDs) for continuous variables and frequencies and percentage relative frequencies for categorical variables.

*The log-transformation was used to compare the means of SGLT-2i users and TZD-users.

ACEi, angiotensin-converting enzyme inhibitor; ALT, alanine aminotransferase; ARB, angiotensin II receptor blocker; ASD, absolute standardized difference; AST, aspartate aminotransferase; BB, beta blocker; BMI, body mass index; CVD, cardiovascular disease; DBP, diastolic blood pressure; eGFR, estimated glomerular filtration rate; FBS, fasting blood sugar; GGT, gamma glutamyl transferase; HDL, high density lipoprotein; HF, heart failure; LDL, low density lipoprotein; MI, myocardial infarction; PAD, peripheral artery disease; SBP, systolic blood pressure; SGLT-2i, sodium glucose co-transporter-2 inhibitor; TZD, thiazolidinedione; WC, waist circumference.
